# New technologies accelerate the exploration of non-coding RNAs in horticultural plants

**DOI:** 10.1038/hortres.2017.31

**Published:** 2017-07-05

**Authors:** Degao Liu, Ritesh Mewalal, Rongbin Hu, Gerald A Tuskan, Xiaohan Yang

**Affiliations:** 1 Biosciences Division, Oak Ridge National Laboratory, Oak Ridge, TN 37831-6422, USA

## Abstract

Non-coding RNAs (ncRNAs), that is, RNAs not translated into proteins, are crucial regulators of a variety of biological processes in plants. While protein-encoding genes have been relatively well-annotated in sequenced genomes, accounting for a small portion of the genome space in plants, the universe of plant ncRNAs is rapidly expanding. Recent advances in experimental and computational technologies have generated a great momentum for discovery and functional characterization of ncRNAs. Here we summarize the classification and known biological functions of plant ncRNAs, review the application of next-generation sequencing (NGS) technology and ribosome profiling technology to ncRNA discovery in horticultural plants and discuss the application of new technologies, especially the new genome-editing tool clustered regularly interspaced short palindromic repeat (CRISPR)/CRISPR-associated protein 9 (Cas9) systems, to functional characterization of plant ncRNAs.

## Introduction

Horticultural plants (for example, such as fruits, vegetables, ornamental trees and flowers, herbs, and tea trees) have been domesticated to satisfy human’s food and aesthetical needs via various forms of hybridization breeding, mutation breeding, and transgenic breeding.^1^ Protein-coding genes related to specific target agricultural trait were chosen as major targets in the early time of transgenic breeding.^2^ Recently, non-coding RNAs (ncRNAs) have been shown to play key roles in the regulation of plant growth, development and response to environmental stresses at either transcriptional or post-transcriptional levels.^3,4^ Thus, ncRNAs are emerging as a spotlighted target materials to accelerate the domestication of horticultural crops.

Though discovery and functional characterization of ncRNAs have been carried out for more than half a century,^5^ their widespread occurrence and myriad functions in various organisms have not been truly appreciated until the post-genomics era. An unexpected finding from the annotation of sequenced genomes is that DNA sequences encoding proteins occupy only a small portion (2–25%) of the genomic space.^6^ The advent of next-generation sequencing (NGS) revolutionized the exploration of ncRNAs, and as a result, many novel ncRNAs have been recently discovered,^7,8^ which were highlighted by the new discovery of circular RNAs (circRNAs).^7, 9,10,11,12^ One of the big challenges in ncRNAs discovery is the determination of the coding potential of RNA sequences. Recent advances in ribosome profiling have shown a great potential for distinguishing between coding and non-coding transcripts and consequently improve the accuracy of ncRNA annotations.^13,14^

Molecular genetics approaches have been applied to functional characterization of ncRNAs via gain-of-function analysis or loss-of-function analysis.^7,15,16^ Precision genome engineering is a powerful tool for functional characterization of ncRNAs. Recently, a platform using RNA-guided engineered nucleases was developed for genome editing. The type II clustered, regularly interspaced, short palindromic repeat, (CRISPR)/CRISPR-associated protein 9 (Cas9) system found naturally occurring in *Streptococcus pyogenes* has been used to obtain rapid and efficient editing of genomes in plant species, and could facilitate the analysis of loss-of-function, gain-of-function and gene expression.^17^

In this review, we describe the classification and known functions of plant ncRNAs. Then, we review the application of NGS and ribosome profiling technology to ncRNAs discovery in horticultural plants, followed by a discussion of the new technologies for functional characterization of ncRNAs.

## Classification and functions of plant ncrnas

**Figure 1:**
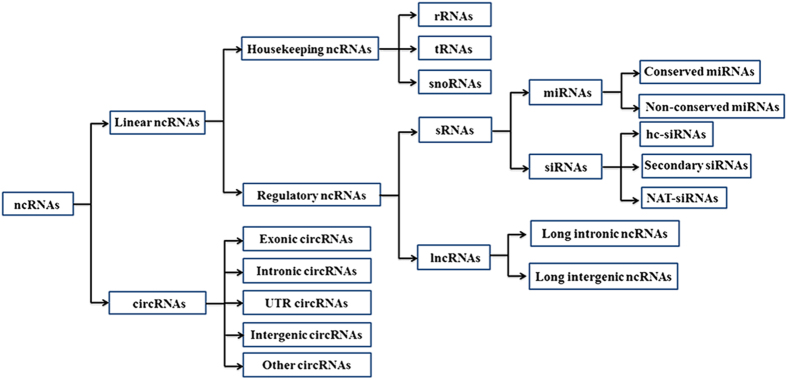
Classification of plant non-coding RNAs (ncRNAs). circRNAs, circular ncRNAs; UTR, untranslated region; rRNAs, ribosomal RNAs; tRNAs, transfer RNAs; snoRNAs, small nucleolar RNAs; sRNAs, small RNAs; lncRNAs, long ncRNAs; miRNAs, microRNAs; siRNAs, small interfering RNAs; hc-siRNAs, heterochromatic siRNAs; NAT-siRNAs, natural antisense transcript siRNAs.

Based on the molecular structure, plant ncRNAs can be classified as linear ncRNAs and circular ncRNAs (circRNAs; Figure 1). The catalog of ncRNAs is currently dominated by linear ncRNAs compared with circRNAs that were just recently discovered as an emerging new class of ncRNAs.^7, 9,10,11,12,18^ On the basis of molecular function, linear ncRNAs can be divided into two categories: (1) housekeeping ncRNAs, including ribosomal RNAs (rRNAs), transfer RNAs (tRNAs) and small nucleolar RNAs (snoRNAs); and (2) regulatory ncRNAs, which can be further divided into two sub-categories: (a) small RNAs (sRNAs), including microRNAs (miRNAs) and small interfering RNAs (siRNAs) and (b) long ncRNAs (lncRNAs), including long intronic ncRNAs and long intergenic ncRNAs.^3,19,20^ On the basis of the genome region from which circRNAs arise, circRNAs can be divided into (1) exonic circRNAs, (2) intronic circRNAs, (3) UTR circRNAs, (4) intergenic circRNAs and (5) other circRNAs deriving from two or more genes (Figure 1).^12^ So far, functional characterization of ncRNAs has focused on sRNAs, lncRNAs and circRNAs. The known biological functions of these three types of ncRNAs are summarized as follows.

### The function of sRNAs

sRNAs are involved in the regulation of plant growth, development and stress response via silencing endogenous gene expression at either transcriptional or post-transcriptional levels.^21,22^

miRNAs, derived from single-stranded hairpin RNAs,^23^ can be classified as conserved miRNAs and non-conserved miRNAs.^24^ Many miRNAs have been characterized from plants, which play important roles in different signaling pathways (Table 1). Usually conserved miRNAs are abundantly expressed, targeting transcription factors that directly regulate gene expression (Table 1). The relationships between conserved miRNAs and their targets have been considered to be stable during the evolution process,^25^ but it was recently reported that the targets of several conserved plant miRNAs (for example, miR396 and miR159) are somewhat flexible.^26,27,28^ In general, non-conserved miRNAs are weakly expressed and have been shown to occur in temporal patterns. Moreover, they are imprecisely processed without tractable targets and thus considered to be randomly evolved with a limited number of biological function.^23^ In addition, primary miRNAs of miR171b of *Medicago truncatula* and miR165a of *Arabidopsis thaliana* have been recently reported to produce peptides, which enhance the accumulation of their corresponding mature miRNAs.^29^

siRNAs, including heterochromatic siRNAs (hc-siRNAs), secondary siRNAs and natural antisense transcript siRNAs (NAT-siRNAs), are derived from Dicer-like (DCL)-catalyzed processing of double-stranded RNA (dsRNA) precursors.^23^ So far, siRNAs have been suggested to play roles in: (1) DNA methylation and chromatin modification mediated by hc-siRNAs,^30^ (2) repression of distinct mRNA targets by *trans*-acting siRNAs^23,31,32,33^ and (3) specific phenotypes, for example, proline accumulation,^34^ fertilization^35^ and bacterial infection,^36^ associated with NAT-siRNAs.

### The function of lncRNAs

lncRNAs are linear ncRNAs of greater than 200 nt in length,^37^ which have been demonstrated to involve in multiple biological processes such as phosphate homeostasis, flowering, photomorphogenesis and fertility (Table 2). The molecular mechanisms underlying the biological function of plant lncRNAs include: (1) processing into shorter ncRNAs for functioning,^38^ (2) acting as the target mimics of miRNAs,^39,40^ (3) repressing histone-modifying activities and direct epigenetic silencing via interaction with specific chromatin domains,^41,42,43,44^ (4) acting as molecular cargo for protein re-localization^45,46^ and (5) post-translational regulation through protein modification and protein–protein interactions.^6^

### The function of circRNAs

Discovery of thousands of circRNAs across a range of plant species have been summarized in other review paper,^47^ and recently demonstrated in horticultural plants, for example, *Solanum lycopersicum*^48^ and *Actinidia chinensis*.^18^ However, little is known about the function of circRNAs in plants. In *Arabidopsis*, Conn *et al.*^49^ reported that the circRNAs derived from exon 6 of the SEPALLATA3 (SEP3) gene can bind strongly to its cognate DNA locus, forming an RNA:DNA hybrid, or R-loop, whereas the linear RNA equivalent bound significantly more weakly to DNA. R-loop formation results in transcriptional pausing, in turn driving floral homeotic phenotypes. The function of circRNAs reported in mammalian may serve as an initial guidance for future studies on the function of plant circRNAs. For example, Hansen *et al.*^50^ reported that circular transcript *ciRS-7* from human and *Sry9* from mouse acts as a ‘molecular sponge’ of miR7 and miR138, respectively. The human circRNA *ITCH* was reported to act as a sponge for miR7, miR17 and miR214, respectively.^51^ Another circRNA *ZNF91* containing 24 miR23 sites, as well as 39 additional sites for miR296, was discovered in mammals.^52^ Zhang *et al.*^9^ showed that an intronic circRNA, *ci-ankrd52*, positively involves in the regulation of RNA polymerase II transcription. Also, exon-intron circRNAs have been shown to enhance the expression of their parental genes in a *cis* configuration.^7^

## Application of new technologies to discovery of ncrnas

**Figure 2:**
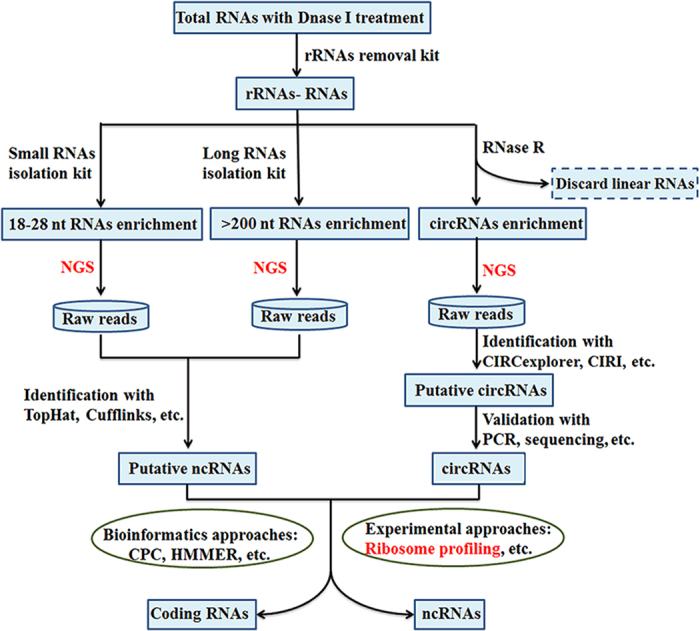
A pipeline for discovery of non-coding RNAs (ncRNAs) in plants. rRNAs, ribosomal RNAs; NGS, next-generation sequencing; CIRI, circular RNA identifier; circRNAs, circular ncRNAs; CPC, coding potential calculator; HMM, hidden markov models.

A variety of experimental approaches have been used for discovering ncRNAs in plants, such as molecular cloning, microarray, next-generation sequencing (NGS), third-generation sequencing,^53^ epitope tagging, mass-spectrometry and ribosome profiling.^54^ These approaches heavily rely on bioinformatics tools, such as TopHat,^55^ Cufflinks,^56^ CIRCexplorer,^57^ CIRI,^58^ CPC^59^ and HMMER,^60^ for the discovery of ncRNAs. Recently, some new computational tools, for example, miRDeep-P,^61^ miRDeepFinder^62^ and miR-PREFeR^63^ were developed for the identification of plant miRNAs, which are often belong to large families with high-sequence similarity among the paralogous members. Moreover, these tools do not necessarily rely on a reference genome and are useful for species-specific ncRNA detection. A pipeline for discovery of ncRNAs in plants is illustrated in Figure 2. Most of above approaches for ncRNA discovery have been discussed in some recent review articles.^7,15^ Currently, more and more horticultural plant genomes and transcriptomes were decoded by third-generation sequencing such as Pacific Biosciences (PacBio, Menlo Park, CA, USA), Illumina Tru-seq Synthetic Long-Read technology (San Diego, CA, USA) and the Oxford Nanopore Technologies sequencing platform (Oxford, UK).^53,64,65,66^ These platforms offer longer read sequencing to facilitate the accurate *de novo* assembly of full-length RNAs without needs for mapping of the transcriptome sequencing reads to the reference genomes. Thus, while still under active development, the third-generation sequencing platforms will definitely accelerate the discovery of ncRNAs and their targets. In combination with appropriate bioinformatics tools such as PLEK,^67^ the ongoing and future efforts for transcriptome sequencing using third-generation sequencing technologies are expected to shed new light on the ncRNA landscape of horticultural plants without reference genomes. Here we focus on two frequently used technologies that offer potential for the discovery and characterization of ncRNAs in horticultural plants: that is, NGS and ribosome profiling.

### NGS as a new powerful tool for the prediction of ncRNAs

The ncRNAs can be identified through the direct detection of the transcribed RNAs.^68^ Initially, direct cloning approach has been used to discover ncRNAs in plants.^69,70^ Subsequently, the hybridization-based microarray technology has been used to discover a large number of ncRNAs in the intergenic regions of *A. thaliana*^71,72^ and rice.^73^ However, the ability of these hybridization-based technologies suffer several limitations such as reduced dynamic range, high false positives^6^ and difficultly defining splice junctions and connecting transcribed regions into transcript models.^74,75^

NGS overcomes the challenges related to microarray technology,^76^ providing a powerful tool for defining the ncRNA domain. For example, miRNAs were previously thought to be dominant members in the sRNAs landscape; however, recent global analysis of plant transcriptomes revealed millions of siRNAs, making them the most abundant class of sRNAs in plants.^77^ More recently, circRNAs were recognized as a large new category of RNAs with thousands of members in animals and plants through high-throughput transcriptome sequencing (RNA-Seq) followed by ncRNA prediction based on RNA-Seq data using new computational algorithms customized for ncRNAs (Figure 2).^7,11,12,57,58^ With advancement of NGS technology, many ncRNAs are being discovered in an expanding list of horticultural plant species (Table 3).

### Ribosome profiling as a new tool for the validation of ncRNA predictions

A key aspect of ncRNA validation is to determine the coding potential of predicted ncRNAs. The length of 18 to 30 nucleotides is the threshold commonly used for the prediction of miRNA^78,79^ whereas the length of greater than 200 nucleotides is often used as the threshold for lncRNAs prediction.^80^ Presence of an open-reading frame (ORF) of at least 100 amino acids (aa) is the threshold commonly used for defining a protein-coding transcript and as such, many important small proteins (<100 aa) were not annotated in plants.^7, 81,82,83^ More recently, a large number of protein sequences have been predicted by translation of the longest ORFs without any further experimental evidence.^74^ It is possible that some of the predicted protein-coding genes, based on an arbitrary ORF length, might be mis-annotated. For example, some well characterized human lncRNAs, such as H19, Hotair, Kcnq1ot1, Meg3 and Xist, contain ORFs of 100 aa or longer.^84^ Most of predicted lncRNAs contain putative ORFs, which may be translated into non-functional proteins or may be unable to be translated at all.^74^

Recently, ribosome profiling, which uses deep sequencing to monitor *in vivo* translation, has shown high potential for the genome-wide examination of protein-coding potential (Figure 2). Ribosome profiling has been used to segregate several hundred small proteins (<100 aa) from predicted lncRNAs in zebrafish and humans.^13,14^ Also，Pamudurti *et al.*^85^ demonstrated that a group of circRNAs was associated with translating ribosomes by performing ribosome profiling from fly heads and found a circRNA generated from the *muscleblind* locus encodes a protein. In *Arabidopsis*, 237 protein-encoding transcripts from the existing compendia of ncRNAs were found based on the ribosome profiling technology.^86,87^ Thus, the ribosome profiling technology can be used as a high-throughput tool for removing false positives in the ncRNAs predictions of horticultural plants.

## Application of new technologies to functional characterization of ncrnas

**Figure 3:**
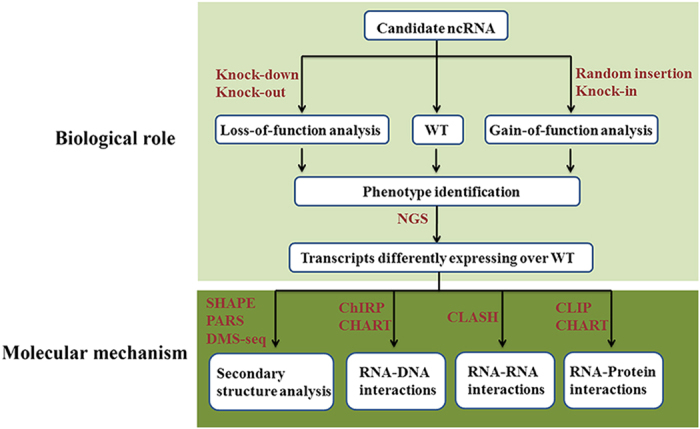
A pipeline for functional characterization of ncRNAs in plants. SHAPE, RNA-selective 2′-hydroxyl acylation and primer extension; PARS, parallel analysis of RNA structure; DMS-seq, dimethyl sulfate-modified RNA for sequencing; CHIRP, chromatin isolation by RNA purification; CHART, capture hybridization analysis of RNA targets, CLASH, crosslinking, ligation, sequencing of hybrids; CLIP, crosslinking immunoprecipitation.

Thanks to the advance in the aforementioned new technologies, the universe of ncRNAs is currently expanding at an increasing rate. However, the biological function of these ncRNAs remains largely unknown.^16^ Various approaches have been developed for functional studies of ncRNAs (Figure 3). The primary goal of functional studies on ncRNAs is to understand the biological processes in which the ncRNAs are involved. To achieve this goal, many researchers have used gain-of-function and loss-of-function mutants for functional characterization of ncRNA genes.^7^ CRISPR/Cas9, a new genome-editing technology, holds great potential for generating knockout and knock-in mutants in plants, as demonstrated in a range of plant species,^17^ and recently demonstrated in horticultural plant species, for example, *Citrus sinensis*,^88^ *Malus pumila*,^89^ *Solanum lycopersicum*^90^ and *Solanum tuberosum*.^91^ Compared with RNA inference (RNAi) that has several limitations such as incomplete gene knock-down and extensive off-target activities, CRISPR/Cas9 technology has the advantage of complete gene knockout with relatively low off-target activities.^92^ In addition, the action of RNAi is restricted in cytoplasm where RNA-induced silencing complexes are located.^93^ However, many ncRNAs have been shown to be localized in the nucleus, which cannot be manipulated in similar manner using RNAi.^68,94^ Thus, CRISPR/Cas9 provides an efficient and effective alternative to RNAi for characterizing the function of ncRNAs. In fact, this new genome-editing technology has been used to knockout several ncRNAs in animals such as humans, mouse, zebrafish,^94–97^ as well as in plants such as soybean.^98^ Once the CRISPR/Cas9-mediated knockout and knock-in mutation is created, the NGS technology, mentioned above, can be used to profile the expression of target transcripts and other downstream genes in the biological pathways (Figure 3).

After identification of the biological roles of ncRNAs, it is important to understand the molecular mechanism underlying these biological roles (Figure 3). Examination of the secondary structure of ncRNAs is informative in studying the function of ncRNAs at the molecular level. Several experimental approaches, such as selective 2′-hydroxyl acylation analyzed by primer extension (SHAPE), parallel analysis of RNA structure (PARS) or dimethyl sulfate-modified RNA for sequencing (DMS-seq), can be used for deciphering of the secondary structure of ncRNAs.^7,15^ To understand where and how the ncRNAs function, chromatin isolation by RNA purification (CHIRP), capture hybridization analysis of RNA targets (CHART), crosslinking, ligation, sequencing of hybrids (CLASH) and crosslinking IP (CLIP) have been developed to detect the interactions between ncRNAs and DNA, RNA or protein.^15,16^ Recently, Shechner *et al.*^99^ used CRISPR/ dCas9, based on a catalytically dead variant of Cas9, to deploy lncRNAs cargos to DNA loci by incorporating the cargo into the sgRNA, thus providing initial insights into the utility of CRISPR/dCas9 for studying the function of ncRNAs. Besides its potential for validating ncRNA prediction, ribosome profiling can also be used to unravel the function of ncRNAs. For example, using ribosome profiling, Guo *et al.*^100^ studied the effects of miRNAs on protein production from their target mRNAs and found that the destabilization of target mRNAs by the miRNAs is the predominant reason for reduced protein output. Similarly, Bazzini *et al.*^101^ studied the impact of miR430 on endogenous mRNAs in zebrafish using ribosome profiling and found that this sRNA reduced translation. These technologies provide new approaches for functional characterization of ncRNAs in horticultural plants.

## Concluding remarks

The discovery and functional characterization of ncRNAs could facilitate the domestication of horticultural plants, resulting in more nutritious, colorful, tasteful, and esthetic fruits, vegetables, and ornamental flowers and trees. While the number of protein-encoding genes is relatively less variable among plants, the ncRNA domain in plants is very dynamic, with increasingly more ncRNA members being discovered and characterized annually. In particular, recent advances in NGS and ribosome profiling technology have offered great potential for expediting the discovery of ncRNAs in horticultural plants. Also, the simplicity, robustness and versatility of the CRISPR/Cas9 systems make such systems attractive for functional characterization of ncRNAs in general and specifically to the process of accelerated domestication in horticultural crops. It is expected that these new technologies will be widely applied in ncRNA research while they become more cost-efficient and more technically mature in the near future.

## Figures and Tables

**Table 1:** Function of miRNAs validated by experiments in plants

*miRNA*	*Conservation*	*Targets*	*Functions*	*References*
miR156	Conserved	SPL	Development	102
miR159	Conserved	MYB, SGN-U567133	Signaling pathway and development	103,28
miR160	Conserved	ARF	Seed germination	104
miR163	Non-conserved	PXMT1, FAMT	Metabolite biosynthesis	105
miR165/166	Conserved	HD-ZIPIII	Leaf and vascular development	106
miR167	Conserved	ARF	Signaling pathway, flower development	107
miR172	Conserved	AP2	Signaling pathway, flower development, stress response	108
miR173	Non-conserved	TAS1, TAS2	Uncharacterized	109
miR319	Conserved	TCP	Flower development	110
miR390	Conserved	TAS	Development	111
miR395	Conserved	Sulfate transporter	Sulfate transport	112
miR396	Conserved	GRF, bHLH74, HaWRKY6	Leaf development, heat tolerance	27,113,26
miR400	Non-conserved	PPR	Heat tolerance	114
miR408	Conserved	Gene coding Copper ion binding protein	Copper homeostasis	115
miR444	Non-conserved	MADS57	Tillering development, nutrition accumulation	115
miR472	Non-conserved	CNLs	Pathogen resistance	116
miR482	Non-conserved	NBS-LRR	Pathogen resistance	117
miR820	Non-conserved	DRM2	Epigenetic silencing	118
miR824	Non-conserved	AGL16	Stomata development, plant flowering	119
miR828	Non-conserved	MYB2	Fiber development	120
miR842/846	Non-conserved	Jacalin lectin	Vegetative storage	121
miR858	Non-conserved	MYB2	Fiber development	120
miR1512	Non-conserved	Gene coding copine-like calmodulin-binding protein	Nodule development	122
miR1863	Non-conserved	Os06g38480	DNA methylation	122
miR4376	Non-conserved	Ca^2+^-ATPase	Flower and fruit development	123
miR5200	Non-conserved	FTL1/2	Flowering initiation	124
miR6019	Non-conserved	NB-LRR/LRR	Pathogen resistance	125
miR6020	Non-conserved	NB-LRR/LRR	Pathogen resistance	125
miR7695	Non-conserved	Nramp6	Pathogen resistance	126

Abbreviations: AGL16, agamous-like 16; AP2, apetala2-like transcription factor; ARF, auxin response factor; bHLH74, basic Helix-Loop-Helix 74; CNLs, coiled-coil nucleotide-binding leucine-rich repeat; DRM2, domains rearranged methyltransferase 2; FTL1/2, flowering locus T-like1/2; FAMT, farnesoic acid carboxyl methyltransferase; GRF, growth regulating factor; HD-ZIPIII, class III homeodomain-leucine zipper transcriptional factor; Nramp6, natural resistance-associated macrophage protein 6; NBS-LRR, nucleotide-binding site-leucine-rich repeat; PPR, pentatricopeptide repeat; SGN-U567133, encoding a protein with unknown function; SPL, squamosa-promoter binding protein-like; TAS, tasiRNA-generating; TCP, teosinte branched1/cycloidea/proliferating cell factor.

**Table 2:** Function of the lncRNAs reported in plants

*lncRNAs*	*Species*	*Biological function*	*Regulation mechanism*	*Refs*
*APOLO*	*Arabidopsis thaliana*	Auxin-controlled development	Chromatin topology	127
*ASCO-lncRNA*	*Arabidopsis thaliana*	Lateral root development	Alternative splicing regulators	127
*asHSFB2a*	*Arabidopsis thaliana*	Gametophytic development	Antisense transcription	128
*cis-NAT*_*PHO1;2*_	*Oryza sativa*	Phosphate homeostasis	Translational enhancer	129
*COLDAIR*	*Arabidopsis thaliana*	Flowering	Histone modification	42
*COOLAIR*	*Arabidopsis thaliana*	Flowering	Histone modification	43
*ENOD40*	*Medicago truncatula, Glycine max*	Nodule development	Protein re-localization	130,131
*HID1*	*Arabidopsis thaliana*	Photomorphogenesis	Association with chromatin	132
*HvCesA6 lnc-NAT*	*Hordeum vulgare*	Cell wall biosynthesis	siRNA precursor	133
*LDMAR*	*Oryza sativa*	Photoperiod-sensitive male sterility	Promoter methylation	134
*XLOC_057324*	*Oryza sativa*	Flowering and sterility	Unknown	135

Abbreviations: APOLO, auxin-regulated promoter loop; ASCO, alternative splicing competitor; asHSFB2a, natural long non-coding antisense RNA of heat stress transcription factor B; PHO1;2, PHOSPHATE1;2; COLDAIR, cold-assisted intronic non-coding RNA; COOLAIR, cold induced long antisense intragenic RNAs; ENOD40, early nodulin 40; HID1, hidden treasure 1; CesA6 lncNAT, natural antisense of CesA6 cellulose synthase gene; IPS1, induced by phosphate starvation 1; LDMAR, long day-specific male-fertility-associated RNA.

**Table 3:** Examples of the application of next-generation sequencing (NGS) technology to ncRNAs discovery in horticultural plants

*Species*	*Type of ncRNAs*	*No. of ncRNAs*	*References*
*Actinidia chinensis*	circRNAs	3582	18
*Arachis hypogaea*	miRNAs	59	136
*Brassica campestris*	miRNAs	131	137
*Brassica napus*	lncRNAs	3181	138
*Cucumis sativus*	lncRNAs	3274	139
*Cucumis sativus*	miRNAs, rRNAs, tRNAs, snoRNAs	1400	140
*Fragaria×ananassa*	miRNAs	190	141
*Phalaenopsis aphrodite*	miRNAs	204	78
*Prunus persica*	lncRNAs	1417	142
*Rosa* sp.	miRNAs	267	143
*Vitis amurensis Rupr.*	miRNAs	232	144
*Solanum lycopersicum*	circRNAs	854	48
*Solanum lycopersicum*	lncRNAs	10774	145
*Solanum tuberosum*	miRNAs	259	146
